# Acute miliary tuberculosis of the pharynx (Isambert disease): case report

**DOI:** 10.11604/pamj.2020.36.249.25158

**Published:** 2020-08-06

**Authors:** Younes Chebraoui, Abdelfattah Aljalil, Mohamed Amine Hanine, Amine Ennouali, Haddou Ammar, Youssef Darouassi, Amine Benjelloun

**Affiliations:** 1 ENT Unit, Avicenne Military Hospital, Marrakech, Morocco,; 2 Pulmonology Unit, Avicenne Military Hospital, Marrakech, Morocco

**Keywords:** Pharyngeal tuberculosis, Isambert, miliary

## Abstract

Miliary pharyngeal tuberculosis or Isambert disease is a rare form of tuberculosis. It can be isolated or more often associated with lung damage. We report the case of a 25-year-old patient referred for deep asthenia with fever, sweating and weight loss, all associated with dysphagia and hearing loss. The pharyngeal examination found an oropharyngeal miliary and an involvement of the cavum. Chest computed tomography (CT) showed excavated lesions suggestive of tuberculosis. The clinical situation clearly improved with anti-tuberculous treatment. Isambert disease is a rare pharyngeal disorder occurring especially at extreme ages or in immunocompromised patients. The contamination is either direct or hematogenous. The pharyngeal examination finds grayish tubers on an often congestive mucosa. The diagnosis is bacteriological and/or histological. Treatment is mainly medical, more rarely surgical. The general assessment aims to find other locations. The miliary pharyngeal tuberculosis is rare and must make seek other locations. The diagnosis is easy and treatment mainly medical. The consequences can be significant.

## Introduction

The acute miliary tuberculosis of the pharynx or Isambert disease is a rare form of pharyngeal tuberculosis and represents less than 1% of the ear, nose and throat (ENT) tuberculosis [1,2]. It is most often associated with lung damage. Isolated forms generally occur at extreme ages or on immunocompromised terrain [3].

## Patient and observation

**Figure 1:**
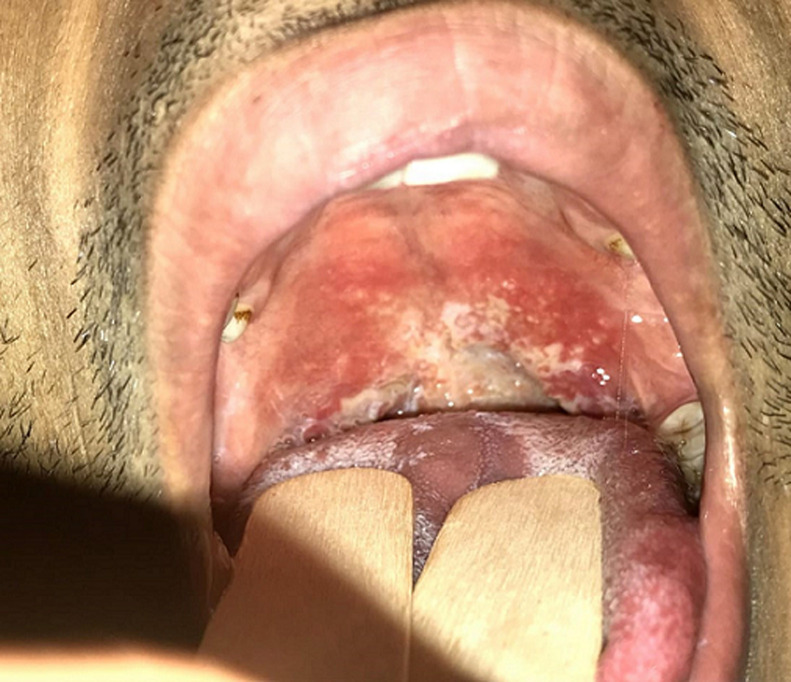
oropharyngeal cavity: multiple non confluent semolina grain granulations of the posterior pharyngeal wall and the veil with purulent secretions

**Figure 2:**
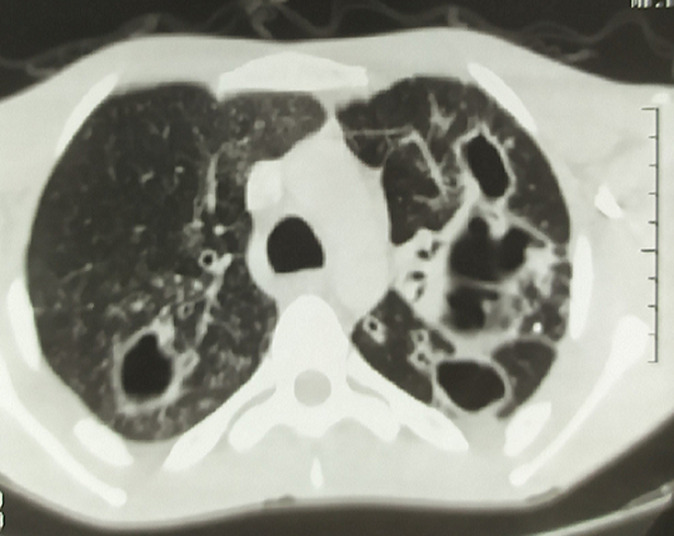
chest CT scan: excavated lesions of the pulmonary apex very suggestive of tuberculosis

A 25-year-old patient with no specific history consulted for dysphagia and bilateral hearing loss with rapid progression for two months associated with fever, night sweats, deep asthenia and eight kilograms weight loss. The examination found an asthenic patient with a 9/6 blood pressure, a 110/mn heart rate and a 39°C fever. Examination of the neck revealed bilateral, firm, sensitive and mobile cervical lymphadenopathies. Oral pharyngeal examination showed multiple, diffuse, non-confluent granulations along the posterior wall of the pharynx and veil, with associated purulent secretions (Figure 1). Endoscopic pharyngolaryngeal examination showed diffuse congestion of the nasal cavity mucosa with a tissue process filling the cavum. The latter had a global purulent appearance. Biopsies were performed in the cavum and oropharyngeal granulations finding an epithelioid granulomatosis suggestive of tuberculosis. The cervico-thoracic CT confirmed the cavum tissue lesion and showed excavated lesions of the two pulmonary fields (Figure 2). Search for Koch bacillus in sputum proved to be positive. A general assessment was carried out, finding no other locations of tuberculosis. The HIV serology was negative. Biology showed an inflammatory syndrome with 15700/mm^3^ white blood cells, 11800/mm^3^ neutrophils and a 190mg/l C-reactive protein (CRP). The rest of biological assessment was normal and showed no obvious immunosuppression. The diagnosis of multifocal tuberculosis was therefore made associating miliary acute tuberculous pharyngitis (Isambert disease) and pulmonary tuberculosis. An anti-tuberculous regimen was started with an excellent clinical and radiological evolution.

## Discussion

Tuberculosis is an infectious disease due to *Mycobacterium tuberculosis*. The pulmonary forms are the most frequent. The ENT forms are less frequent and largely dominated by lymph node tuberculosis. The pharyngeal lesions occur mainly on immunocompromised terrain in developed countries because the pharynx is a zone relatively immune to the bacillus. This is explained by regular salivary cleansing, the presence of saprophytic germs, antagonism of the striated musculature against bacillary invasion, a thick pharyngeal epithelium and local pH [2-4]. In countries endemic for tuberculosis, these forms are more frequent and often associated with lung or other visceral involvement as in the present case. Pharyngeal contamination occurs either by hematogenous route in the pulmonary miliary forms or more often by rising contaminated bronchial secretions [1,2]. Miliary tuberculosis of the acute pharynx or Isambert disease is a rare form of pharyngeal tuberculosis and represents less than 1% of ENT forms [1,2]. Isolated affections occur mainly on immunocompromised terrain or at extreme ages [3]. The nasopharyngeal lesions are the most frequent. This infection is associated with high fever, profuse sweating and significant weight loss. Several symptoms have been described such as headache, sore throat, cough, tinnitus, hearing loss, dysphagia, odynophagia, postnasal discharge and other signs [3]. The pharyngeal examination finds a granulation of grayish or semi-transparent “semolina grains” or “fish eggs” resting on a pale or congestive mucosa [3,5]. At a more advanced stage, the confluence of these granulations on the veil and the pillars gives potentially hemorrhagic ulcers with protruding edges, resting on a hard base [5]. The mobility of the palate can be altered with an edematous uvula.

Differential diagnosis is usually made with herpetic angina or buccopharyngeal candidiasis [5]. The larynx can also be congestive with tuberculous granulations. The presence of cervical lymphadenopathy is almost usual in this affection. The ENT and general examination must of course be exhaustive and screen for all other possible locations of the disease. The imaging is not specific. The pharyngeal and cervical CT examination eliminates an aggressive lesion, a pharyngeal extension of cervical spine tuberculosis and gives details on the lymphatic network, especially cervical lymphadenopathies [5,6]. The biological inflammatory syndrome is usual with elevated CRP, leukopenia sometimes leukocytosis, rarely lymphopenia. Inflammatory anemia is also frequent [7]. The presence of Koch bacillus by direct examination, culture, or polymerase chain reaction (PCR) can be demonstrated by pharyngeal swab, collection of sputum and lymph node or pharyngeal biopsy. Histological analysis also confirms the diagnosis by showing epithelioid granulomatosis with or without caseous necrosis [7]. The search for other tuberculosis foci involves systematic chest imaging and must then be referred by the clinical signs. Testing for Koch bacillus in the urine is unnecessary in the absence of macro or microscopic hematuria. The treatment is medical, combining four medications (rifampicin, isoniazid, ethambutol and pyrazinamide) according to the WHO protocol (6-month regimen). Surgery is reserved for major fistulized lymph node lesions, threatening tonsillary forms as well as severe sequelae: synechiae, perforations, stenosis, fibrous scars with functional and aesthetic damage [8,9]. Apart from these complications, prognosis is good and healing is usual.

## Conclusion

Miliary pharyngeal tuberculosis is a rare condition and occurs mainly on immunocompromised terrain. Its presence must seek other locations, ENT and pulmonary in particular. Its treatment is mainly medical, surgery is reserved for threatening or serious sequelae forms.
